# Moult of overwintering Wood Warblers *Phylloscopus sibilatrix* in an annual-cycle perspective

**DOI:** 10.1007/s10336-021-01859-z

**Published:** 2021-02-15

**Authors:** Crinan Jarrett, Luke L. Powell, Tabe T. Regine Claire, Melanie Tchoumbou, Barbara Helm

**Affiliations:** 1 Institute of Biodiversity, Animal Health and Comparative Medicine, University of Glasgow, Glasgow, G12 8QQ UK; 2 Department of Animal Biology, Vector Borne Diseases Laboratory of the Applied Biology and Ecology Research Unit (VBID-RUBEA), University of Dschang, Dschang, Cameroon; 3 GELIFES, Groningen Institute for Evolutionary Life Sciences, University of Groningen, Groningen, The Netherlands; 4 Biodiversity Initiative, Houghton, MI 49913 USA; 5 Department of Biosciences, Durham University, Stockton Road, Durham, DH13LE UK

**Keywords:** Migrant, Moult, *Phylloscopus sibilatrix*, Sub-saharan Africa, Cocoa agroforestry, Timing programme

## Abstract

Wood Warblers, an Afro-Palearctic migrant species, are declining steadily in Europe likely due to mortality outside their breeding grounds. However, little is known about their overwintering, and records about the sensitive life-cycle stage of moult in Africa are practically absent. To fill this gap, we report on moult of Wood Warblers captured over two winters (January–February) in 2019–2020 in Cameroon. We caught 14 individuals, of which 12 were monitored for flight feather moult. All inspected individuals showed advanced stages of flight feather renewal. Despite low sample sizes, Underhill-Zucchini moult models aptly explained variation in primary and secondary moult (*R*^2^ = 0.61). Estimated moult onset date was 26 December, completion date was 25 February, and moult duration was 61 days. These findings fit well with experimental data on the annual cycle and the timing of recently published migration tracks of Wood Warblers. Jointly, the data suggest that moult timing is set by an internal programme, which enables Wood Warblers to organise their multi-stage migration such that they reach suitable moulting habitat in time, and can depart in time with a fresh plumage for the breeding grounds. In our study, moult occurred during the peak of the dry season, which in Cameroon nonetheless shows high relative humidity. During our mist-netting on 28 cocoa plantations of varying shade cover, Wood Warblers were caught on 6 farms whose canopies were comparatively open. These data suggest that the birds encounter in Cameroon relatively stable climatic conditions for moult, and do not measurably prefer closed-canopy forests. Our findings are important, because successful moult increases survival prospects and because moult needs to be safely embedded in a migratory life cycle. Hence, information on moult timing and location is essential for identifying year-round vulnerabilities of Wood Warblers.

## Abstract (de)

**Mauser von überwinternden Waldlaubsängern** ***Phylloscopus sibilatrix*** **im Kontext des Jahreszyklus**

Waldlaubsänger nehmen in weiten Teilen Europas stetig ab, vermutlich aufgrund von Sterblichkeit außerhalb der Brutgebiete. Dennoch ist über die Überwinterung dieser afro-paläarktischen Langstreckenzieher wenig bekannt. Insbesondere fehlen Daten zur Mauser, die zum großen Teil in den afrikanischen Überwinterungsgebieten stattfindet. Zur Klärung dieser verletzlichen Lebenszyklusphase präsentieren wir hier Daten zur Mauser von Waldlaubsängern von Januar und Februar 2019-2020 in Kamerun. Von 14 Individuen, die bei standardisierten Beringungsstudien kontrolliert wurden, wurden 12 eingehend auf Mauser überprüft. Diese Vögel zeigten fortgeschrittene Stadien der Großgefiedermauser. Trotz niedriger Stichprobengröße konnten Underhill-Zucchini-Mausermodelle die Variation in der Hand- und Armschwingenmauser weitgehend erklären (R^2^ = 0.61). Der geschätzte Beginn der Mauser war der 26. Dezember, Mauserende war der 25. Februar, und die Dauer betrug 61 Tage. Diese Ergebnisse passen gut zu experimentellen Daten zum Jahreszyklus und zu kürzlich veröffentlichten Informationen zu den Zugwegen des Waldlaubsängers. Insgesamt legen die Daten nahe, dass der Zeitpunkt der Mauser durch ein endogenes Programm gesteuert wird. Dadurch können Waldlaubsänger ihre mehrstufige Zugsaison so organisieren, dass sie rechtzeitig zur Mauser geeignete Habitate erreichen, und im Anschluss daran mit frisch erneuertem Gefieder in die Brutgebiete ziehen. In unserer Untersuchung fand die Mauser zum Höhepunkt der Trockenzeit statt. Da jedoch in dieser Zeit in Kamerun die relative Luftfeuchtigkeit vergleichsweise hoch bleibt, können die Vögel unter weitgehend stabilen klimatischen Bedingungen mausern. Die Beringungsstudien fanden auf 28 Kakaoplantagen mit unterschiedlich stark geschlossenem Kronendach statt. Alle 14 Waldlaubsänger wurden auf 6 Plantagen gefangen, deren Kronendach vergleichsweise offen war. Offensichtlich zeigen Waldlaubsänger zu dieser Zeit keine messbare Bevorzugung von geschlossenen Kronen. Diese Ergebnisse sind wichtig, weil eine erfolgreiche Mauser die Überlebenschancen erhöht, und weil die Mauser sicher in den Migrationszyklus und Jahreslauf eingebettet werden muss. Informationen zu Zeit und Ort der Mauser sind unerlässlich, damit Gefährdungspotenziale während des gesamten Jahreszyklus erkannt werden können.

## Introduction

**Fig. 1:**
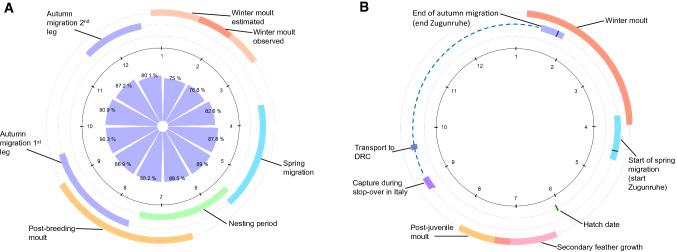
Annual cycle of Wood Warblers **a** in the wild and **b** under experimental conditions. **a** Combined field data from our study on winter moult, and from literature sources on all other annual-cycle stages (Cramp 1988; Snow and Perrins 1998). Red shades indicate remige moult, blue shades migration, and green shades breeding. Innermost cycle shows long-term monthly means of relative humidity, accessed from https://power.larc.nasa.gov. **b** Combined captivity data from two studies by Gwinner (1968,1969), where one investigated adult migration and overwintering, and the other juvenile development; colour shades as in **a** Dotted line on the migration cycle indicates sustained migratory restlessness. Note that data are given for 3 of the 4 individuals studied by Gwinner (1968) in Democratic Republic of Congo (DRC). The 4th bird is omitted, because it moulted no remiges

Wood Warblers *(Phylloscopus sibilatrix)* are small songbirds that breed at mid to high latitudes from western Europe to western Asia, and winter near the equator in central and West Africa (Curry-Lindahl 1981; Hobson et al. 2014; Tøttrup et al. 2018). Populations of this long-distance Afro-Palearctic migrant have been declining steadily in Europe, with a 37% decrease between 1980 and 2015 (PECBMS 2018). The causes of these declines are yet unclear; they could be related to changes occurring in the breeding grounds, wintering grounds, or during migration. Given the highly dynamic and seasonal life-history of Wood Warblers, we require information on their whole annual cycle to better understand the pressures this species is facing. In general, birds arrive at their breeding grounds around May, depart for a complex journey in early August after a partial post-breeding moult, and undergo a complete moult at their wintering sites, which they leave in March (for details see below, Fig. 1; Cramp 1988; Jenni and Winkler 2020a).

Understanding the timing and location (both geographically and in terms of habitat characteristics) of Wood Warblers' life-cycle events is important for conservation, for example to investigate links between rapid forest loss in the Afrotropics and population declines (Hobson et al. 2014; Mallord et al. 2018). Breeding season events are relatively well documented and indicate a clear preference for closed-canopy forest habitat (Maziarz and Wesołowski 2010; Mallord et al. 2016; Huber et al. 2017; Lerche‐Jørgensen et al. 2019). Studies from Europe show no or weak evidence of changes in breeding habitat characteristics, phenological mismatch or nest mortality driving population declines (Maziarz and Wesołowski 2010; Mallord et al. 2012, 2017; Lerche‐Jørgensen et al. 2019). In contrast, our knowledge of Wood Warbler locations, life-cycle events and ecology outside their breeding grounds is limited. Migration routes are poorly known, but recently published tracking data support a multi-step journey (Curry-Lindahl 1981; Tøttrup et al. 2018; Jiguet et al. 2019). Habitat preferences are unclear outside the breeding season (Mallord et al. 2016; Awa et al. 2018; Lerche‐Jørgensen et al. 2019). Based on their breeding behaviour, Wood Warblers are expected to prefer forest habitats, but evidence is mixed as they have also been detected in savannah and agricultural land at staging and wintering sites (Hobson et al. 2014; Mallord et al. 2016, 2018; Weisshaupt and Rodríguez-Pérez 2017; Awa et al. 2018; Lerche‐Jørgensen et al. 2019). Importantly, within their wintering habitats Wood Warblers undergo moult, a critical life-cycle stage, which is almost completely undocumented (Jenni and Winkler 2020a).

Moult, which in passerines occurs at least once a year, is costly in terms of energy and time (Jenni and Winkler 2020a, b). Consequently, the intensity (quantified from simultaneously replaced feathers) and timing of moults are strongly linked to fitness. Moult intensity is associated with (a) the energetic and nutritional requirements of moult, and thereby also quality of the resultant plumage, and (b) the reduction in plumage functions (e.g. flight, manoeuvrability, thermoregulation). Vulnerability, for example to predation, increases particularly during primary moult (Jenni and Winkler, 2020a). Intensity of moult changes over its course, with a peak during mid-moult, and is often lower when time pressure is relaxed, for example during wintering (Jenni and Winkler 2020a). Conversely, several studies showed that feather quality can be reduced if birds moult under time-pressure (Hall and Fransson 2000).

Further indication that moult is critical for long-term fitness comes from studies of moult timing. Some resident birds in the Afrotropics, notably Common Bulbuls *Pycnonotus barbatus* in Nigeria, show stronger seasonality in moult than in breeding, which suggests a strong selection to match timing of moult to resources (Nwaogu et al. 2019). This is corroborated by data showing that the timing of moult in Bulbuls reflects a local gradient of timing of rains (Nwaogu and Cresswell 2020). For long-distance migrants, the timing of moult is particularly important, because replacement of feathers needs to be embedded into a migratory life style. To avoid major overlaps between flight feather moult and migration, moult is best timed to phases in the annual cycle when birds can remain stationary in areas with sufficient resources (Weber et al. 2005; Jenni and Winkler 2020a).

Habitat is an important factor affecting success of moult (Jenni and Winkler 2020a, b), because moult can be heavily influenced by the availability of resources in the environment. For example, in Nigerian Common Bulbuls moult timing and progress was enhanced by both rains and a fruit diet (Nwaogu et al. 2020; Nwaogu and Cresswell 2020). Within other species, aridity has been associated with slow moult progress (Borras et al. 2003), and moult differed between urban and non-urban populations (Hope et al. 2016). To better target actions for conservation of Wood Warblers, it is, therefore, important to understand their habitat association during moult. Changes in habitat, such as the land-use conversion from forest to agriculture, may severely influence the availability of their insect food supplies (Watt et al. 1997).

Because of a need for a stationary phase, moult also acts as a constraint to the spatio-temporal course of migration. Trans-Saharan migrants can moult in late-summer on the breeding grounds, anytime during wintering, or both (Pearson 1973; Jenni and Winkler 2020a). The choice of moult strategy is linked to several factors including distance of migration, relative food availability in breeding and non-breeding grounds, and time available between breeding and departure for migration (Jenni and Winkler 2020a). Many examples indicate evolutionary lability in the timing of moult in long-distance migrants, for example in a recent comparative study of Common Whitethroats *Sylvia communis* (Remisiewicz et al. 2019) and in comparative studies of the genus Phylloscopus, including Wood Warblers (Gwinner 1968, 1969). Given the spatio-temporal complexity of their annual cycles, many long-distance migrants use inherited, circannual programmes to time their activities including migration and moult (Curry-Lindahl 1981; Gwinner 1996; Åkesson and Helm 2020). Such endogenous programmes have been demonstrated in several taxa of Phylloscopus warblers including Wood Warblers (Gwinner 1968), where annual-cycle activities were recorded to persist in birds flown to Bukavu (Democratic Republic of Congo, DRC). However, robust field data from free-living individuals are needed to examine how timing programmes are implemented in the wild.

Here, we, therefore, present data on locations, timing and moult details of Wood Warblers in West Africa. Our study is aimed not only to fill the gap of knowledge on the annual cycle, but also to contribute to conservation of this species. Thus, we carried out standardized mist-netting in differently farmed cocoa agroforests in southern Cameroon. Agroforestry systems such as cocoa farms offer a range of habitats, from intensively managed farms with open canopies, to low intensity farms with thick forest-like canopies (Jarrett et al. in press; Tscharntke et al. 2011). We carefully selected farms that represented a gradient of canopy cover. Our gradient allowed us to test the hypothesis that overwintering Wood Warblers select closed-canopy habitats, similar to their breeding habitat.

We aimed to describe (a) the intensity and (b) the timing of moult in overwintering Wood Warblers, and (c) to investigate whether Wood Warblers selected closed-canopy cocoa farms during moult.

## Methods

**Fig. 2:**
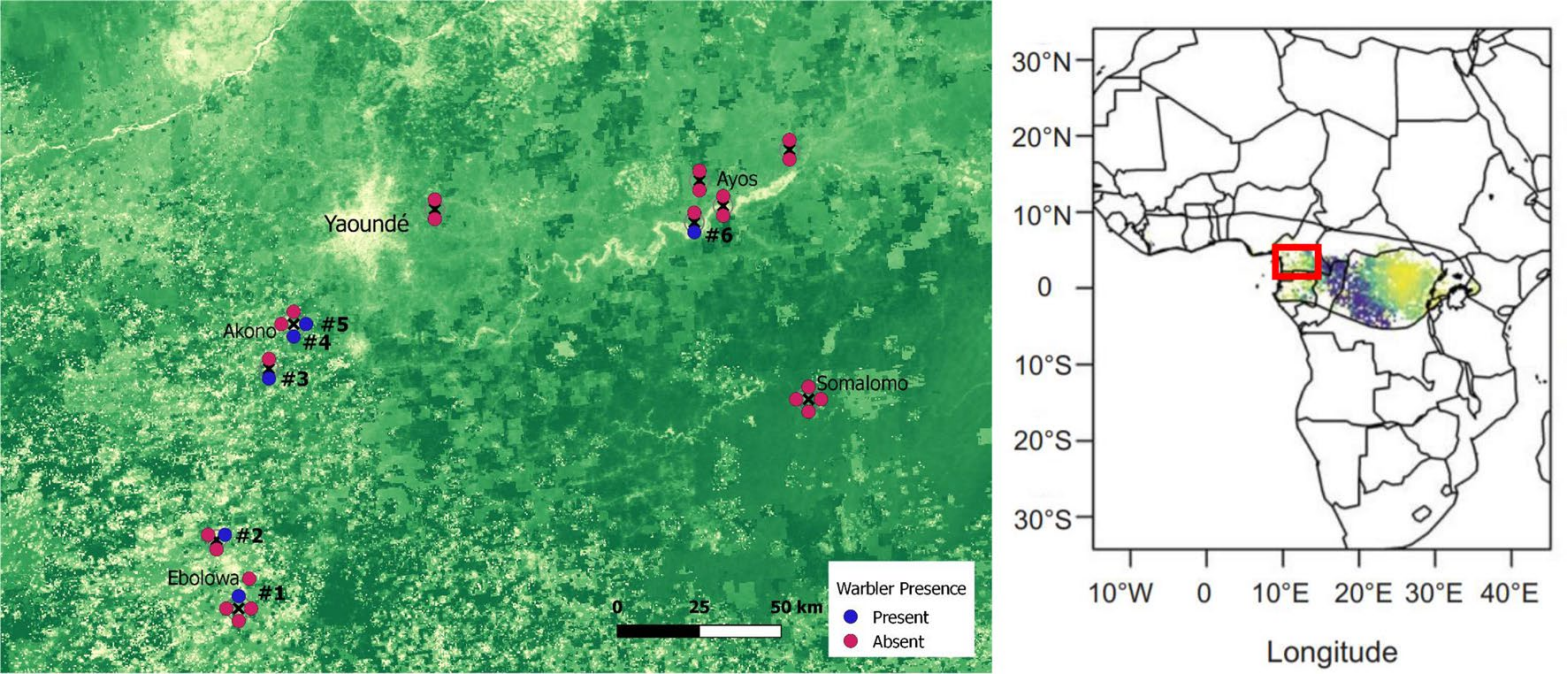
Cocoa farms sampled in 2019–2020 (*n* = 28) in Southern Cameroon with dots coloured according to warbler presence or absence (left), and the same locations superimposed on the predictive map of Wood Warbler moult origins from Hobson et al (2014; right panel). The base map shows eMODIS Normalised Difference Vegetation Index (NDVI; October 2018; accessed from https://earlywarning.usgs.gov/), where darker green indicates more forested areas. The numbers indicate the order in which farms where visited (see ‘Farm ID’ in Table 1)

We carried out mist-netting in January—February 2019–20 in cocoa farms in southern Cameroon (2° 49′—3° 51′N, 11° 6′—12° 25′E; Fig. 2; Jarrett et al. in press). The sequence and timing of visits are detailed in Fig. 2 and Table 1. Cocoa farms were minimum 1.5 ha and at least 500 m apart. At each farm, we set up 20 12 × 3.2 m mist-nets and opened them for 6 h per site (06:20–12:20). Wood Warblers were rare on these farms, representing 0.9% of all captures. We captured 14 Wood Warblers (3 in 2019; 11 in 2020), of which 13 were examined for body moult and 12 additionally for wing moult. All birds were lean (fat score 0–1 in EURING system; EURING 2010).

**Table 1:**
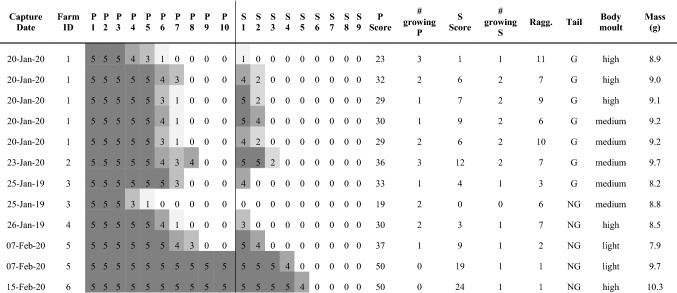
Moult scores of Wood Warblers (*n* = 12) caught in cocoa farms in Southern Cameroon in 2019–2020

For farm locations, see 'Farm ID' and Fig. 1. P1-P10 give the moult score for each primary feather and S1-S9 give the moult score for each secondary feather. 'P score' and 'S score' are the moult scores across primary and secondary feathers, respectively. '# growing P' and '# number S' give the number of growing primary and secondary feathers respectively. 'Ragg.' gives raggedness values, which refer to primaries and secondaries. 'Tail' indicates whether birds' tail was growing ('G') or not growing ('NG')

We assessed moult of all wing remiges (wing flight feathers), consisting of 10 primaries and 9 secondaries, including the innermost secondaries that are sometimes called tertials (EURING 2010). We numbered remiges in their natural moulting sequence, i.e. primaries from mid-wing to tip (descendently), whereas secondaries were numbered from mid-wing to body (ascendently). We scored moult of remiges (Redfern and Clark 2001) from 0 to 5, where 0 represents an old feather, 5 a completely renewed feather, and scores 1–4 a growing feather in moult. Primary moult scores represent the sum of scores across 10 primaries, thus the maximum primary moult score is 50, and secondary moult scores are the sum of scores over 9 secondaries, thus the maximum is 45 (EURING 2010). From these scores, we quantified moult intensity as the number of simultaneously growing primaries and 6 outermost secondaries, as well as the gap left by these growing feathers (raggedness), for all birds with primary moult scores of 20–40 (Bensch et al. 1991). Tail feathers were scored as either growing or not growing. Body moult, if present, was scored as ‘light’ (< 1/3 feather tracts in moult), ‘medium’ (1/3–2/3 feather tracts in moult) or ‘heavy’ (> 2/3 feather tracts in moult). We weighed all birds and scored furcular fat following the EURING code (EURING 2010).

Analyses of the data were carried out in the statistical software environment R version 3.5.1 (R Core Team 2019). Despite our low sample size, we provide estimates of remige moult timing and duration because data from Wood Warblers are so scarce. We first linearized moult data with a conversion formula of remiges to feather mass derived from Common Whitethroats (Remisiewicz et al. 2019). We then predicted duration, start and end of moult using the Underhill and Zucchini moult model (Underhill and Zucchini 1988) implemented in the package *moult* (Erni et al. 2013). We used a type 4 model, which is suited for data that are representative of the part of the population in moult and post-moult, even if early stages of moult are lacking. We acknowledge that because of the small number of individuals, caution is advised when interpreting the results.

To better understand seasonal constraints in Wood Warblers, we downloaded data on monthly relative humidity from https://power.larc.nasa.gov/ for a location in the centre of our sampling sites, for years 1981–2019 (spanning the phenology data shown in Fig. 1a).

To test whether agroforestry type influenced presence of Wood Warblers, we considered shade cover as an indication of how intensely the farm was managed. To measure shade cover, we took photographs at ten locations in each farm, spaced out by 24 m and at least 50 m from farm edge. We took photographs using a camera with a fish-eye lens on an extendable pole (12 m). Using the software ImageJ (Schneider et al. 2012), we converted the photographs to black and white, and then calculated the percentage of black (vegetation) in each photograph. The shade cover value for a farm was a mean of the ten pictures. Shade cover values ranged from 19.6% in the most intensively managed farm to 98.7% in the least intensively managed farms. To compare characteristics of farms where warblers were present or absent, we used a generalised linear model with a binomial distribution and shade cover as explanatory variable. We compared this model with the null model using a likelihood-ratio test (LRT) to test significance. We report the chi-squared value and the *p* value associated to the LRT.

## Results

**Fig. 3:**
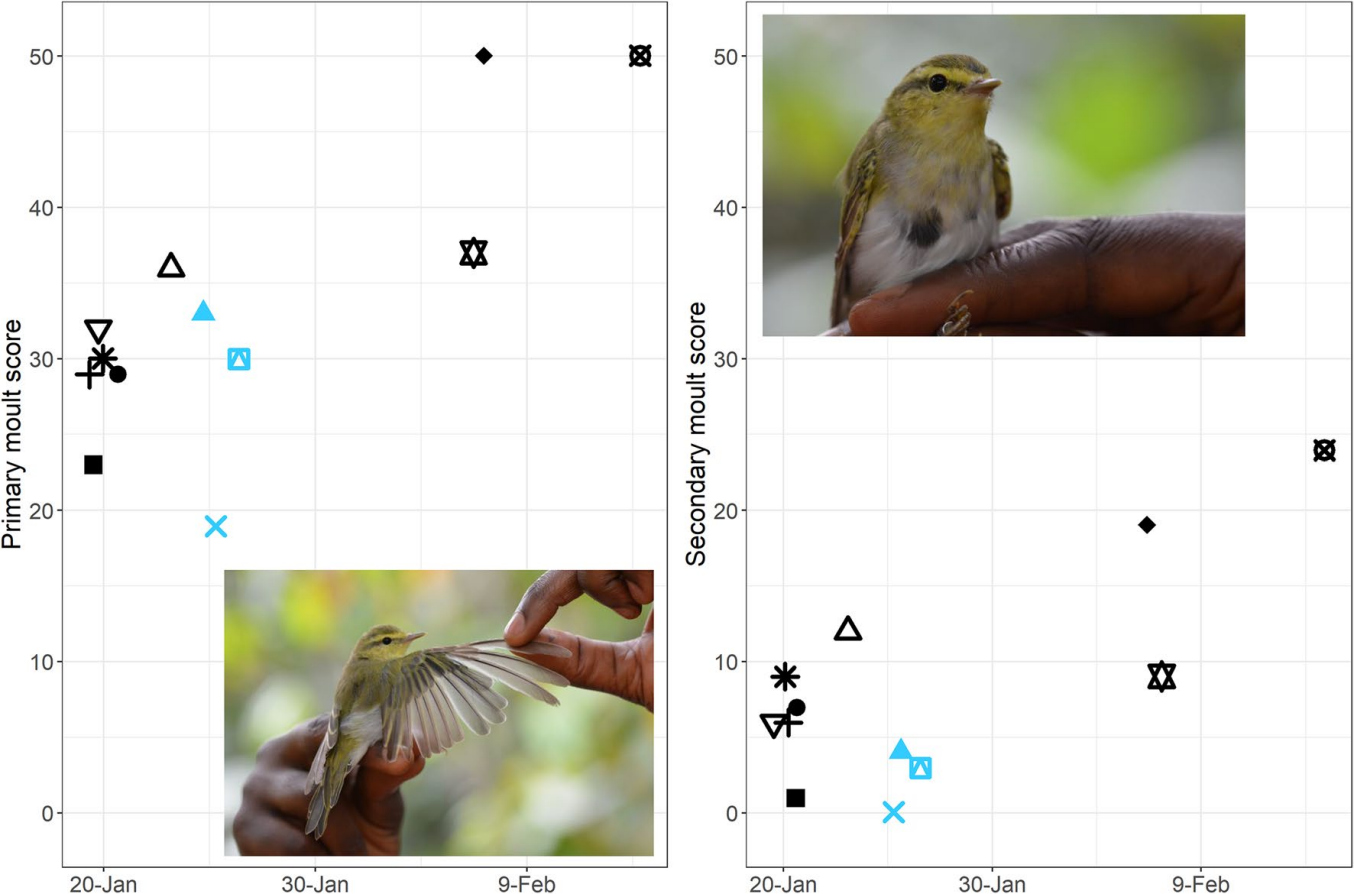
Moult score of the 10 primaries (left) and 9 secondaries (right) against date. Each shape represents an individual bird, and colour corresponds to year (blue = 2019, black = 2020). Photographs: Wood Warblers in Cameroonian cocoa farms, taken by CJ

**Fig. 4:**
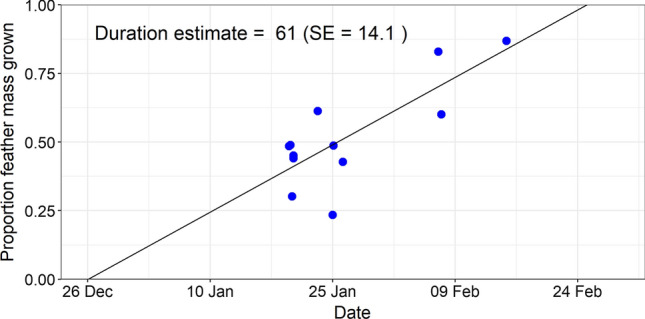
Raw data for proportion feather mass grown (primaries and secondaries) with date, and regression line based on Underhill-Zucchini model (R^2^ = 0.61). Mean start date was estimated to be 26 December (SE = 7.6), and moult duration to be 61 days (SE = 14.1), giving a finish date of 25 February

All inspected individuals showed active body moult (*n* = 13) and advanced flight feather moult (*n* = 12; Fig. 3; Table 1). Tail growth was observed in all individuals caught before 25 January, and not later. The Wood Warblers we caught showed primary moult between mid-way and completion, and secondary moult from onset to mid-way; in both feather tracts moult progressed with date (Fig. 3). Mean start date for moult was estimated to be 26 December (SE = 7.6), with a variation of 6.8 days (SE = 3.8). The duration of primary and secondary moult was estimated at 61 days (SE = 14.1), ending on 25 February (Fig. 4). The number of moulting primary feathers decreased significantly with date (intercept = 3.6, slope = − 0.08, *R*^2^ = 0.42, *p* = 0.01). During core primary moult (primary score 20–40; *n* = 9), the mean number of growing primaries was 1.8 (range = 1–3) and mean raggedness was 6.9 (range = 2–11). Body mass of captured Wood Warblers ranged from 7.9 to 10.3 g (mean = 9.1 g). Of 13 birds scored, 8 had no fat (score 0), 4 had trace of fat (score 1) and 1 had up to 1/3 of tracheal pit obscured by fat (score 2). Moult of our birds in Cameroon overlapped with the driest season of the year, but mean monthly relative humidity was still high (≥ 75%, Fig. 1a).

Of the 28 cocoa farms visited, we detected Wood Warblers in 6 (Fig. 2). The mean shade cover of the farms in which warblers were detected was 54.1% (± 31.6), non-significantly lower than the 71.8% (± 24.8) in the farms where warblers were not found (*n* = 28, *χ*^*2*^ = 2.03, *p* = 0.15).

## Discussion

Our findings, although based on low sample sizes, help to fill in an important gap in the understanding of the annual cycle of Wood Warblers, the complete moult at wintering grounds (Fig. 1). Wood Warblers studied over 2 years moulted during January and February, possibly during their second major stop-over in Africa, and prior to spring departure in mid-March (Fig. 1). Our estimated moult start in late December fits well with the sparse previous field records from the species (Stresemann 1955; Cramp 1988; Jenni and Winkler 2020a) and the captivity data by Gwinner (1968).

The Wood Warblers captured on Cameroonian cocoa plantations showed moderate moult intensity compared to other species. A moult gap of up to three primaries would generate considerable flight impediment, especially if the gap is close to the outer edge of the wing (Jenni and Winkler 2020a). Still, raggedness in our birds (range 2–11) was far lower than that of passerine migrants moulting on breeding grounds in Finland (70°N; range 14–22; Haukioja, 1971), and in the lower third of those moulting on winter grounds in Ghana (11°N; range 3–24; Bensch et al. 1991). Nonetheless, our estimated remige moult duration (~ 61 days) was similar to estimates of primary moult duration from other Afro-tropical migrant songbirds. Although some species in Ghana moulted more rapidly (Bensch et al. 1991), most estimates from European migrants across Africa were of similar or longer duration for primary moult, while our estimates included secondaries (65–80 days; Pearson 1973; Aidley and Wilkinson 1987; Salewski et al. 2004). Compared to our Cameroonian Wood Warblers, congeneric Willow warblers (*Phylloscopus trochilus*) moulted more rapidly on breeding grounds across Europe (range of means 31–53 days for primary moult; Underhill et al. 1992), but spent similar time on pre-breeding moult in Africa (48–68 days, Guinea Bissau; Underhill et al. 1992).

Moult during overwintering is common in sub-Saharan Africa, and becomes the dominant strategy for migrants staying southward from the equator (Jenni and Winkler 2020a). However, migrants differ in the timing and intensity of this moult. There is a general trend to moult toward the end of the rainy season, when resources are at a peak (Jenni and Winkler 2020a). Differences between migrants have thus been linked to the timing of rains in their African moulting grounds, where the early dry season north of the equator is thought to favour early and rapid moult (Bensch et al. 1991; Jenni and Winkler 2020a). Our findings roughly fit with this pattern: in our study area close to the equator, Wood Warblers moult relatively late compared with birds overwintering further north, which moult in November–January (Aidley and Wilkinson 1987; Bensch et al. 1991).

However, the moult period for our birds coincided with the driest period of the year in south-eastern Cameroon. This timing could be explained in several ways. First, there are benefits of moulting shortly before departure for spring migration, including a fresh plumage for the return journey and breeding season (Jenni and Winkler 2020a). This strategy, however, comes with the risk of having to depart with incomplete remige moult, as occasionally observed in Wood Warblers (Jenni and Winkler 2020a). Second, a complex multi-step journey in autumn may result in a late arrival in Cameroon, leaving little alternative but to moult during the dry season (Tøttrup et al. 2018; Fig. 1a). Third, in southern Cameroon, costs may be eased, as relative humidity remains high around the year (Fig. 1a). At our sites, during the dry season only some of the trees lose their foliage, and like in other parts of West Africa, the dry season coincides with flowering of many tree species, in turn attracting insects (Nwaogu et al. 2019; Jenni and Winkler 2020a). Indeed, preliminary insect sampling in our study-sites suggested that insect communities fluctuate seasonally; overall, we saw an increase of Hemiptera and Hymenoptera in the dry season, whereas in the wet season, Diptera were more abundant (Powell et al., unpublished data). This third explanation is also supported by the mean body mass of captured birds, which was only marginally lower than body mass of birds in their breeding grounds (range 9.5–11 g; Jiguet et al. 2019; Tøttrup et al. 2018).

For Wood Warblers, benefits of a fresh wing for spring migration and breeding combined with relatively stable resources may have thus selected for moult in West Africa (Hobson et al. 2014) in December–February, with implications for the birds' entire life cycle (Fig. 1a). That Wood Warblers largely rely on an inherited time programme for their migration (Curry-Lindahl 1981) is supported by the excellent fit of our data on moult and recently published migration tracks (Tøttrup et al. 2018; Jiguet et al. 2019) with Gwinner's (1968) seminal experimental studies on circannual rhythms. Alongside 10 Willow Warblers, Gwinner transported 4 Wood Warblers in September to DRC (Bukavu, 2°S, 28°E) and subsequently recorded annual-cycle events in outdoor and indoor aviaries (Fig. 1b). The Wood Warblers had been captured at an Italian stop-over site in late August. Thereafter in DRC they continuously displayed migratory restlessness (Zugunruhe) until mid-January, ceasing it only shortly after they initiated remige moult in early January (Gwinner 1968). Moult extended until late March, when the birds re-initiated migratory restlessness. In Fig. 1b, these data are combined with data from a second study by Gwinner, in which he detailed the timing of juvenile and post-juvenile plumage development of captive Wood Warblers (Gwinner 1969).

Gwinner's (1968) moult data overlap with our observations, except for longer moult duration in the captive birds. The migratory restlessness data can be compared to migration data from two recent tracking studies. A Danish-ringed Wood Warbler left Italy after a stop-over in mid-August and reached Sudan a month later, where it remained for 2 months (Tøttrup et al. 2018). From mid-November, it moved westward until it reached Côte d’Ivoire on 20 December, where it was recorded until equipment failure in mid-January. Additional records from British Wood Warblers (Jiguet et al. 2019) also support notions that Wood Warblers combine a first migration leg to central Africa with a second migration leg to West Africa, from where they depart in early April by desert crossings (Tøttrup et al. 2018; Jiguet et al. 2019). If these tracks are representative, moult timing of Wood Warblers might be constrained to occur in a narrow time window within a complex spatio-temporal programme of activities within Africa (Fig. 1). This scenario is supported by migratory restlessness, which only ceased during the time window of moult (Gwinner 1968). The otherwise continuous expression of migratory restlessness may well indicate a readiness to facultatively move in response to environmental conditions, at times other than during the moulting window (Helms 1963; Terrill and Ohmart 1984). Such an inherited spatio-temporal programme would highlight the importance of knowing and protecting suitable moulting habitat. Our study is amongst the first to report the presence of Wood Warblers in cocoa agroforestry systems in Africa (but see Awa et al., 2018). Contrary to expectations, our findings suggest that Wood Warblers did not select for more closed-canopied cocoa farms. We consider two possible interpretations. First, we cannot exclude that our result arises from detectability bias, due to potentially greater likelihood of catching birds in mist-nets amongst low vegetation (Remsen and Good 1996). Second, Wood Warblers may be relatively flexible and resilient in habitat choice outside the breeding season, as indicated also by other reports (Mallord et al. 2016, 2018; Weisshaupt and Rodríguez-Pérez 2017; Awa et al. 2018; Lerche‐Jørgensen et al. 2019). African cocoa farms are known to harbour diverse bird communities, including forest birds in farms with a thick canopy (Jarrett et al. in press). Additionally, at our study sites in southern Cameroon, blocks of forest were still available. Such refuges into which the birds could move might locally mitigate against negative effects of agroforestry. Although the quality of regrown plumage is unknown, the occurrence of flight feather moult in all examined Wood Warblers in cocoa farms indicates that these Cameroonian agroforestry habitats, perhaps in conjunction with neighbouring habitats, were sufficiently resource-rich to meet the high energetic demands of moult.

Overall, our study contributes to a more complete understanding of the annual cycle of Wood Warblers. By combining existing knowledge of their annual programme with our novel records, we highlight the complexity and consistency of this species’ life-style. We conclude that, for the conservation of long-distance migrants such as Wood Warblers, we must pay special attention to timing, location and habitat of winter moult in the context of their yearly schedule.

## Data Availability

Data supporting the results is archived at https://doi.org/10.6084/m9.figshare.13258991.v1.

## Code availability

Code supporting the results is archived at https://doi.org/10.6084/m9.figshare.13258991.v1.
